# CFD-Based and Experimental Hydrodynamic Characterization of the Single-Use Bioreactor Xcellerex^TM^ XDR-10

**DOI:** 10.3390/bioengineering9010022

**Published:** 2022-01-08

**Authors:** Diana Kreitmayer, Srikanth R. Gopireddy, Tomomi Matsuura, Yuichi Aki, Yuta Katayama, Takuya Nakano, Takuma Eguchi, Hirofumi Kakihara, Koichi Nonaka, Thomas Profitlich, Nora A. Urbanetz, Eva Gutheil

**Affiliations:** 1 Interdisciplinary Center for Scientific Computing, Heidelberg University, 69120 Heidelberg, Germany; diana.kreitmayer@iwr.uni-heidelberg.de; 2 Pharmaceutical Development, Daiichi-Sankyo Europe GmbH, 85276 Pfaffenhofen, Germany; srikanth.gopireddy@daiichi-sankyo.eu (S.R.G.); thomas.profitlich@daiichi-sankyo.eu (T.P.); nora.urbanetz@daiichi-sankyo.eu (N.A.U.); 3 Biologics Technology Research Laboratories, Biologics Division, Daiichi-Sankyo Co., Ltd., Fukushima 971-8183, Japan; matsuura.tomomi.g2@daiichisankyo.co.jp (T.M.); aki.yuichi.ha@daiichisankyo.co.jp (Y.A.); katayama.yuta.nt@daiichisankyo.co.jp (Y.K.); nakano.takuya.zu@daiichisankyo.co.jp (T.N.); eguchi.takuma.vd@daiichisankyo.co.jp (T.E.); kakihara.hirofumi.d8@daiichisankyo.co.jp (H.K.); nonaka.koichi.k2@daiichisankyo.co.jp (K.N.)

**Keywords:** Xcellerex^TM^ XDR-10, single-use bioreactor, mixing time, oxygen mass transfer coefficient, surface vortex formation, computational fluid dynamics, one-way Euler–Lagrange simulation, Euler–Euler simulation

## Abstract

Understanding the hydrodynamic conditions in bioreactors is of utmost importance for the selection of operating conditions during cell culture process development. In the present study, the two-phase flow in the lab-scale single-use bioreactor Xcellerex${}^{\mathrm{TM}}$ XDR-10 is characterized for working volumes from 4.5 L to 10 L, impeller speeds from 40 rpm to 360 rpm, and sparging with two different microporous spargers at rates from 0.02 L min${}^{-1}$ to 0.5 L min${}^{-1}$. The numerical simulations are performed with the one-way coupled Euler–Lagrange and the Euler–Euler models. The results of the agitated liquid height, the mixing time, and the volumetric oxygen mass transfer coefficient are compared to experiments. For the unbaffled XDR-10, strong surface vortex formation is found for the maximum impeller speed. To support the selection of suitable impeller speeds for cell cultivation, the surface vortex formation, the average turbulence energy dissipation rate, the hydrodynamic stress, and the mixing time are analyzed and discussed. Surface vortex formation is observed for the maximum impeller speed. Mixing times are below 30 s across all conditions, and volumetric oxygen mass transfer coefficients of up to 22.1 h${}^{-1}$ are found. The XDR-10 provides hydrodynamic conditions which are well suited for the cultivation of animal cells, despite the unusual design of a single bottom-mounted impeller and an unbaffled cultivation bioreactor.

## 1. Introduction

Single-use bioreactors have become popular because of their lower investment costs and reduced downtime between cultivation runs [1] compared to standard stainless steel multi-use bioreactors. However, the geometry of single-use reactors significantly deviates from classical stainless-steel reactors in the positioning of the impeller(s) and the lack of baffles [1,2]. For this type of geometry, there is less experience in optimization of the operating conditions compared to the classical reactors. Therefore, computational fluid dynamics (CFD) simulations are important to investigate the hydrodynamics in single-use bioreactors [3,4].

Out of the Xcellerex${}^{\mathrm{TM}}$ XDR bioreactors [5], the Xcellerex${}^{\mathrm{TM}}$ XDR-10 is the smallest one. This cylindrical bioreactor has a flat bottom, a centered, bottom-mounted pitched-blade impeller, and the maximum working volume is 10 L. Four spargers are integrated, two drilled-hole spargers with five openings of 0.5 and 1.0 mm diameters, respectively, and two microporous spargers consisting of sintered disks of 1.2 cm in diameter and with possible pore diameters of 2 μm and 20 μm, respectively. All sparger openings are integrated into the plate on which the impeller is fixed. This positioning of the sparger openings below the impeller is equal in all Xcellerex${}^{\mathrm{TM}}$ XDR bioreactors [5]. In the series of the Xcellerex${}^{\mathrm{TM}}$ XDR bioreactors, the smallest reactors, XDR-10 and XDR-50, have a flat bottom and a centered impeller, whereas all larger Xcellerex${}^{\mathrm{TM}}$ XDR reactors have a curved bottom and an off-centered impeller. Thus, the geometric similarity, which is a typical scale-up criterion, is not fulfilled in the series of Xcellerex${}^{\mathrm{TM}}$ XDR bioreactors, and a careful evaluation of the hydrodynamics in those reactors is required.

In stirred-tank bioreactors, homogenization is achieved by the rotational motion of the impeller, and oxygen is transferred to the cultivation medium by sparging air or oxygen. Consequently, the flow type can be described as a mechanically agitated liquid with rising dispersed bubbles. Possible modeling approaches for this kind of system are the Euler–Lagrange model, where discrete bubbles are tracked, and the Euler–Euler formulation, where the liquid and the gas phases are in Eulerian coordinates with different volume fractions [6,7,8,9]. In the one-way coupled Euler–Lagrange approach, only the effect of the continuous liquid phase on the gas bubbles is considered, and in the formulation with two-way coupling, the effect of the dispersed bubbles on the continuous phase is also considered. The present study concerns the one-way coupled Euler–Lagrange formulation.

Mishra et al. [10] investigated the XDR-10; they considered the bubble size distribution and studied the volumetric oxygen mass transfer coefficient for the 20 μm sparger. The present study concerns the microporous spargers with focus on the 2 μm pore size since they lead to a higher oxygen mass transfer compared to the 20 μm size. Mishra et al. [10] used empirical correlations to determine the mixing time and not with a detailed computational simulation. In the present study, Euler–Lagrange simulations are performed with the commercial software MixIT [11], which is commonly used for the characterization of industrial stirred tank reactors and for which only one-way coupling is available. Even though this approach neglects the impact of bubbles on the liquid, its low computational cost and fast screening of potential process conditions make it attractive for engineering applications. The full coupling of both phases is considered in the Euler–Euler simulations, which are performed with the open-source code OpenFOAM [12].

A total of eight simulations considering different working volumes, impeller speeds, sparging rates, and sparger types are carried out with both methods. The computed liquid and bubble flow fields are investigated. The simulated surface vortex formation is compared with new experimental data of the change in liquid height at the vessel wall. The risk of cell damage is analyzed considering the simulation results for the vortex formation [13,14,15], the average turbulence energy dissipation rate [16], and the hydrodynamic stress [17,18]. Furthermore, the mixing time without and with sparging and the volumetric oxygen mass transfer coefficient are measured and compared with the numerical results.

## 2. Material and Methods

First, the setup of the bioreactor and the selected test conditions are presented, followed by the details of the experimental methods. Furthermore, the governing equations of the models and their numerical implementation are summarized.

### 2.1. Configuration of the Bioreactor and Test Conditions

**Figure 1:**
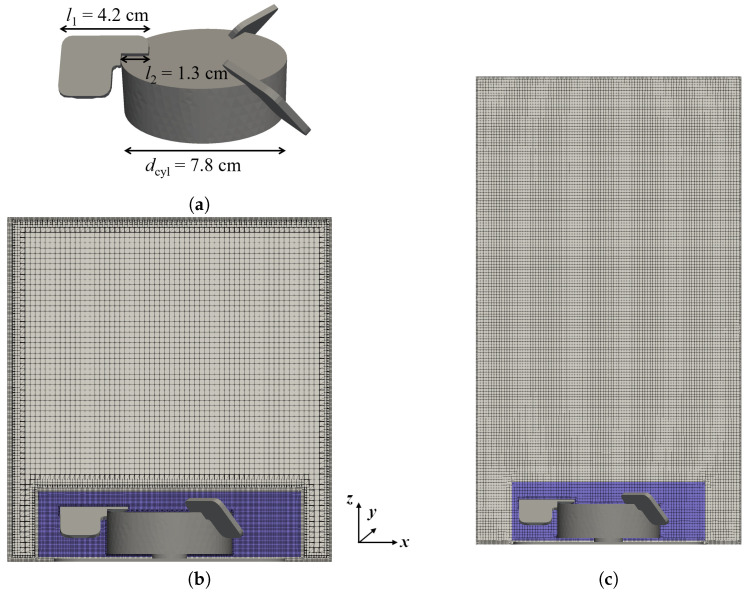
(**a**) Impeller dimensions. (**b**) Vertical cut of the Euler–Lagrange grid. (**c**) Vertical cut of the Euler–Euler grid. The blue areas show the regions to which the rotating reference frame is applied.

The Xcellerex${}^{\mathrm{TM}}$ XDR-10 consists of a flexible, single-use bag with a hexagonal cross-section in which the spargers and the magnetically driven impeller are already integrated. The bag is placed in a solid, cylindrical outer shell to which it is deemed to perfectly conform to and accordingly, a cylindrical vessel shape is considered in simulations. This is in contrast to the recent study by Mishra et al. [10], where a hexagonal cross-section is used for the same XDR-10 bioreactor. The impeller has three pitched blades, angled at 40, see Figure 1a, and the impeller diameter $d_{\mathrm{imp}}=d_{\mathrm{cyl}}+2\left(l_{1}-l_{2}\right)$ is 13.6 cm. Four disk-shaped sparger openings are available, two microporous and two drilled hole spargers.

In the present study, the pore sizes of 2 μm or 20 μm of the microporous spargers are considered, while the drilled-hole spargers are omitted as they produce larger bubbles that result in a lower volumetric oxygen mass transfer coefficient. The average bubble diameter across the whole bioreactor is dominated by the sparger where they are formed, and through possible break-up and coalescence during the rise of the bubbles through the reactor. Mishra et al. [10] report bubble Sauter mean diameters between 0.1 mm and 2 mm across the different regions of the bioreactor for the 20 μm sparger based on their population balance modeling results. Correlations from the literature [19,20] provide initial bubbles sizes of 0.8 mm to 1.3 mm for the 20 μm sparger. According to these consideration of the overall size range and the expected initial bubble size, a bubble diameter of 1 mm is selected for the 20 μm sparger in the present study.

For the 2 μm sparger, no information on the overall size range is available, and initial bubble diameters of 0.4 to 0.6 mm are calculated using different correlations [10,19,20]. Since smaller bubbles are moving at lower velocity compared to large bubbles because of their lower momentum, a longer residence time and a larger gas hold-up and coalescence rate are expected, and the average bubble size across the bioreactor will be larger than the initial bubble size. Accordingly, a bubble diameter of 0.8 mm is selected for the 2 μm sparger, which is slightly larger than the initial diameter range and slightly lower than the mean bubble diameter the 20 μm sparger.

All test conditions are summarized in Table 1. The operating conditions are chosen by increasing or reducing a single parameter at a time, while keeping all others at the intermediate level (see condition 4—base case). The 2 μm pore size is selected as the standard setting. For condition 8, in which the maximum impeller speed is evaluated, simulations are performed at 360 rpm while for the corresponding experiments, 350 rpm is used because at a rotational speed of 360 rpm, no stable conditions could be achieved due to mechanical instability.

### 2.2. Experimental Methods

This subsection provides details on the experiments for measuring the mixing time, the volumetric oxygen mass transfer coefficient, and the change in liquid height. For the mixing time and the volumetric oxygen mass transfer coefficient, measurements are taken in triplicate.

Mixing time measurements are performed with the iodine de-colorization method [21]. The bioreactor is filled with tap water and is colorized by adding 4 mL starch solution (10 g L ${}^{-1}$ soluble starch and 2 g L ${}^{-1}$ benzoic acid) per 1 L working volume and 0.1 mL iodine solution (400 g L ${}^{-1}$ potassium iodine and 127 g L ${}^{-1}$ iodine) per 1 L working volume. The addition of 0.1 mL thiosulfate tracer (166 g L ${}^{-1}$ 1N sodium thiosulfate) per 1 L working volume leads to decolorization. The mixing time is taken with a stopwatch as the time from tracer addition until complete decolorization.

The volumetric oxygen mass transfer coefficient $k_{L}a$ is measured with the dynamic method [22] at 37 C in the previously described model medium [23]. Initially, the dissolved oxygen is removed from the liquid by sparging with nitrogen. Then sparging with oxygen is started and the increase in oxygen tension is monitored over time. To remove accumulated nitrogen gas from the head space, an additional air flow of 0.1 L min${}^{-1}$ is applied directly to the head space. The $k_{L}a$ is determined as the negative slope of the temporal evolution of the natural logarithm of the dissolved oxygen tension

(1) $$\ln\left(\frac{{\mathrm{DO}}^{*}-{\mathrm{DO}}_{t}}{{\mathrm{DO}}^{*}-{\mathrm{DO}}_{0}}\right)=-k_{L}a\left(t-t_{0}\right),$$

where ${\mathrm{DO}}^{*}$, ${\mathrm{DO}}_{0}$, and ${\mathrm{DO}}_{t}$ are the dissolved oxygen tension at saturation, at the initial time $t_{0}$ and at time *t*, respectively. To correct for the impact of the head space airflow on the $k_{L}a$, the $k_{L}a$ with only head space airflow is measured separately and subtracted from the total $k_{L}a$ for all test conditions.

The change in liquid height due to sparging and stirring for all conditions is measured with a ruler as the difference in liquid height at the vessel wall before stirring and sparging is started and after the liquid height has stabilized again.

### 2.3. Mathematical Models

The study concerns a liquid flow at low Mach number with bubbles that are considered to remain spherically symmetric throughout the simulation.

For the one-way coupled Euler–Lagrange simulations, the effect of the gas bubbles on the liquid phase is neglected, and the liquid flow is described by the incompressible Reynolds-averaged Navier–Stokes equations using the standard *k*-ε model [24]

(2) ∇·u=0

(3) $$\frac{\partial u}{\partial t}+\nabla \cdot \left(u⊗u\right)=-\nabla \left(\frac{p}{\rho }\right)+\nabla \cdot \left(\nu_{\mathrm{eff}}\left(\nabla u+\nabla u^{T}-\frac{2}{3}Iu\right)-\frac{2}{3}Ik\right)$$

(4) $$\frac{\partial k}{\partial t}+\nabla \cdot \left(ku\right)=\nabla \cdot \left(\left(\nu +\frac{\nu_{t}}{\sigma_{k}}\right)\nabla k\right)+G_{k}-\varepsilon$$

(5) $$\frac{\partial \varepsilon }{\partial t}+\nabla \cdot \left(\varepsilon u\right)=\nabla \cdot \left(\left(\nu +\frac{\nu_{t}}{\sigma_{\varepsilon }}\right)\nabla \varepsilon \right)+C_{1}\frac{\varepsilon }{k}G_{k}-C_{2}\frac{\varepsilon^{2}}{k},$$

where ρ and u are the liquid density and velocity, respectively, *p* denotes pressure. The effective kinematic viscosity $\nu_{\mathrm{eff}}=\nu +\nu_{t}$ is the sum of molecular, ν, and the turbulence, $\nu_{t}=C_{\mu }k^{2}/\varepsilon$, viscosities, where *k* and ε are the turbulence kinetic energy and its energy dissipation rate. I denotes the identity matrix. The model coefficients $\sigma_{k},\sigma_{\varepsilon },C_{1}$, $C_{2}$, and $C_{\mu }$ are 1.0, 1.2, 1.44, 1.92, and 0.09 [24]. $G_{k}$ is the turbulence production term.

The bubble trajectories are modeled by Lagrangian particle tracking

(6) $$V_{b}\rho_{g}\frac{\partial v}{\partial t}=\frac{3}{4}C_{D}V_{b}\frac{\rho }{d_{b}}\left(u-v\right)\left|u-v\right|+V_{b}g\left(\rho_{g}-\rho \right)+V_{b}\rho \frac{Du}{Dt},$$

where $V_{b}$, $d_{b}$, and v are the bubble volume, the bubble diameter, and the bubble velocity, respectively, and $\rho_{g}$, **g**, and $\frac{D}{Dt}$ denote the gas density, the gravitational acceleration, and the substantial derivative. The first term on the right-hand side is the drag force, and the second and third terms are the gravitational and the pressure gradient forces. The drag coefficient, $C_{D}$, is calculated as [25]

(7) CD=0.424ifReb>100024Reb1+16Reb2/3ifReb≤1000

where the bubble Reynolds number is Reb=db|u−v|ρμ.

The properties of the liquid phase are taken from the experiment where the density ρ = 1010.8 kg m${}^{-3}$ and the dynamic viscosity of the cell culture medium, μ = 0.001126 Pa s. The gas phase is pure oxygen with a density and a dynamic viscosity of 1.29 kg m${}^{-3}$ and 2.1 × 10${}^{-5}$ Pa s [26], respectively.

One-way coupled Euler–Lagrange simulations are run with MixIT [11]. For all conditions, a flat top boundary is used to which a slip condition is applied. In the first step, single-phase steady simulations are run for 20,000 iterations after which a constant impeller torque and average velocity are achieved. In the second step, Lagrangian tracking of the bubbles is performed based on the liquid flow results.

For the Euler–Euler approach, the continuous phase equations are solved, and the interphase momentum transfer is included by a source term in the momentum equations [23]. In the Euler–Euler simulations, the turbulence is modeled with the mixture *k*-ε model [27]. The drag coefficient is described by the Schiller–Naumann model [28]. Additionally, the virtual mass force $F_{\mathrm{VM}}=C_{\mathrm{VM}}V_{b}\rho \left(Du/Dt-Dv/Dt\right)$ is considered with a virtual mass coefficient $C_{\mathrm{VM}}$ of 0.5 [6,9,23].

The Euler–Euler simulations are performed with OpenFOAM version 7 [12] using the reactingTwoPhaseEulerFoam solver. The Euler–Euler formulation allows for the inclusion of the head-space of the bioreactor. The simulations are run until steadiness is reached, i.e., less than 3% of variation in the gas hold-up, in the volume-average liquid velocity, and in the impeller, torque is reached.

The grid generation is performed with the OpenFOAM [12] meshing tool snappyHexMesh. For the Euler–Lagrange (EL) simulations, a separate grid for each working volume is created using the pre-configured settings of MixIT [11]. In addition to the grid refinement at the wall boundaries, the meshing strategy implemented by MixIT also includes grid refinement of the multiple reference frame (MRF) region surrounding the impeller. This leads to a high local grid density, and about 50% of the 3.5 × 10${}^{6}$ control volumes of the 7 L volume are located in the MRF region. For the Euler–Euler (EE) simulations, a mesh of the complete bioreactor is created. Grid refinement is only applied to the boundaries and for resolving the edges of the MRF region. This significantly reduces the number of control volumes to 1.9 × 10${}^{6}$, and only about 9% of control volumes are located in the MRF region. For both approaches, grid independence is tested with single-phase steady-state simulations, and the numerical grids presented in Figure 1 are found to be sufficiently refined based on less than 2% differences in the average liquid velocity and the impeller torque between the selected and the finest grid.

To quantify the possible differences in the numerical solutions introduced by the differences in the grids, the Euler–Euler grid is cut to the same working volume as the Euler–Lagrange grid, and Euler–Lagrange simulations are performed with both grids using OpenFOAM, which assures that the same discretization of the equations and the identical numerical solution procedures are used. The volume-average velocity magnitude differs by about 7%.

The mixing process is simulated by solving for the transport of the normalized concentration, *c*, of a passive tracer

(8) $$\frac{\partial (\alpha_{l}c)}{\partial t}+\nabla \cdot \left(\alpha_{l}c\;u\right)-\nabla \cdot \left(\alpha_{l}\left[D+\frac{\nu_{t}}{{\mathrm{Sc}}_{t}}\right]\nabla c\right)=0.$$

*D* is the molecular diffusivity of the tracer, and ${\mathrm{Sc}}_{t}$ denotes the turbulent Schmidt number which has the standard value 0.7 [4]. To determine the mixing time, the change in the tracer concentration is evaluated at five different monitor positions in different regions of the reactor: two located directly below the liquid surface close to the wall on opposite side, one in the center at an intermediate height, another close to the reactor wall, and another one at the bottom of the reactor close to the wall. The mixing time is evaluated, when the relative change in the tracer concentration at all monitor position is less than 3%.

Evaluation of the specific oxygen mass transfer coefficient $k_{L}a$ from simulations is evaluated following the eddy cell model of Lamont and Scott [29]

(9) $$k_{L}a=0.4\sqrt{D_{O_{2}}}{\left(\frac{\varepsilon }{\nu }\right)}^{0.25}a,$$

where the gas–liquid exchange area per unit of liquid volume is

(10) $$a=\frac{6\alpha_{g}}{d_{b}}$$

and $D_{O_{2}}$ = 3.0 × 10${}^{-9}$ m${}^{2}$s${}^{-1}$ is the diffusion coefficient of oxygen in water at 37 C [30] and the kinematic viscosity is ν = 1.11 × 10${}^{-6}$ m${}^{2}$s${}^{-1}$.

In the Euler–Lagrange approach, the average gas volume fraction $\alpha_{g}=Qt_{r}/V$ where *Q*, $t_{r}$, and *V* denote the sparging rate, the average bubble residence time, and the working volume. In the Euler–Euler model, the gas volume fraction $\alpha_{g}$ is a simulation result.

In the Euler–Lagrange simulations, the surface vortex depth

(11) Δh=Δp/(ρ|g|)

is calculated from the pressure difference Δp between the wall and the center of the top boundary, which is considered to correlate to the hydrostatic pressure difference Δp that is found between the lowest and highest liquid level of the surface vortex. In the Euler–Euler simulations, the surface vortex depth is evaluated from the shape of the air–liquid interface represented by the iso-surface of the volume fraction $\alpha_{g}=1-\alpha_{l}=0.5$. The surface vortex depth and the increase in liquid height at the wall are determined as the maximum liquid extension in the vertical direction and its difference to the unagitated liquid height, respectively.

## 3. Results and Discussion

The results are presented and discussed in terms of the characteristics of the stirred-tank bioreactor. First, the numerical results of the liquid flow pattern, the bubble dispersion, the surface vortex formation, and the hydrodynamic stress are presented. Then, the experimental and simulated mixing times and volumetric oxygen mass transfer coefficients are compared and discussed.

### 3.1. Liquid Velocity and Bubble Dispersion

**Figure 2:**
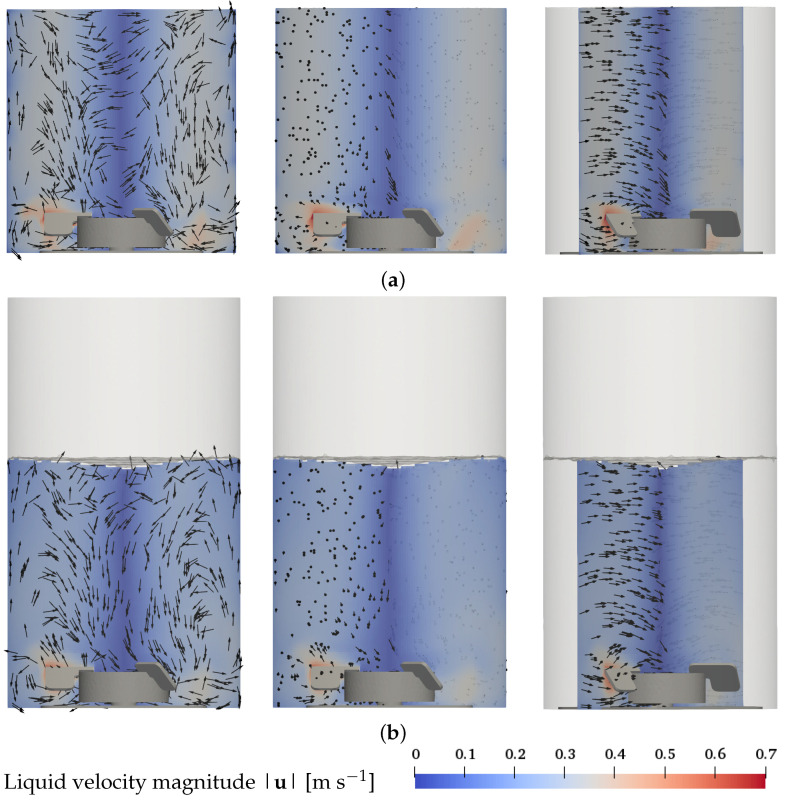
Liquid flow pattern for the base condition 4 (7 L, 100 rpm, 0.25 L min${}^{-1}$, 2 μm) in the *x*-*z* plane plane through the center of the vessel. Left hand side: front view, arrows show the *x*-*z*-velocity direction. Center and right hand side: front and angled views, resp., arrows show the three-dimensional velocity direction. (**a**) Euler–Lagrange results; (**b**) Euler–Euler results.

The numerical results of the liquid flow field for the base case, condition 4 (7 L, 100 rpm, 0.25 L min${}^{-1}$, 2 μm) are presented in Figure 2. The liquid flow is plotted on a vertical cut plane through the center of the vessel and the part of the impeller in front of the plane is shown for orientation. In both modeling approaches, a dominant rotational motion due to the stirring is visible. The secondary structure of radial and axial velocities shows two flow recirculation zones: a small recirculation zone with a high velocity resides below the impeller blades, and a large recirculation zone with a lower velocity is located above the impeller blades. These recirculation zones are best visible in the plot of the planar *x*-*z*-velocity direction on the left-hand sides of Figure 2a for the EL simulations and Figure 2b for the EE simulations. Due to the anti-clockwise rotational motion of the liquid, the three-dimensional velocity direction has a dominant tangential component in the direction normal to the cut plane, which makes it difficult to identify the recirculation zones from that perspective, see center of Figure 2a,b. The tangential component is best visible at the angled view shown in center and right parts of Figure 2a,b.

A similar general flow pattern of the two recirculation zones is found across the different operating conditions, which are not shown. An increase in the liquid volume leads to a higher upper recirculation zone at a similar position in the bioreactor. A reduction of the impeller speed results in a lower liquid velocity. For the Euler–Euler approach at the highest impeller speed, a strong surface vortex is observed, deforming the shape of the upper recirculation zone in addition to the increase in the liquid velocity. Surface vortices, in general, cannot be captured in Euler–Lagrange simulations because the liquid surface is represented as a flat top boundary.

**Figure 3:**
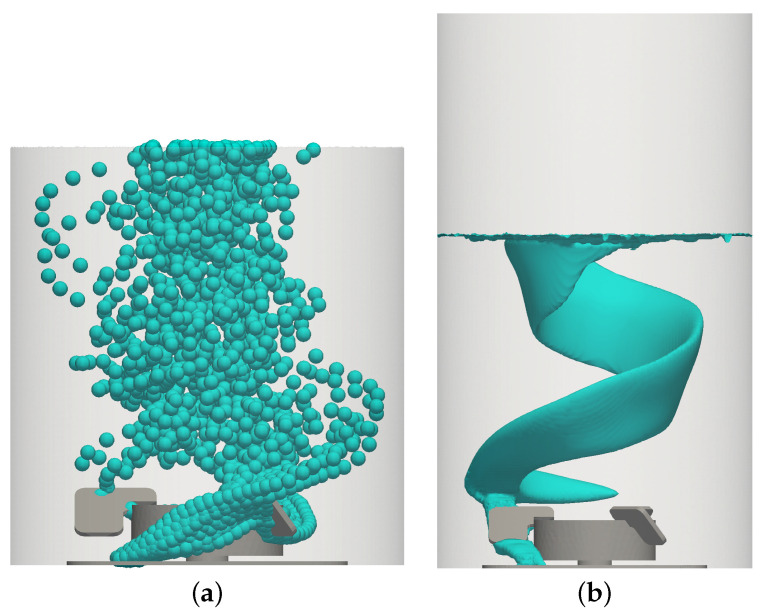
Bubble dispersion for the base condition 4 (7 L, 100 rpm, 0.25 L min${}^{-1}$, 2 μm). (**a**) Euler–Lagrange results; (**b**) Euler–Euler results, $\alpha_{g}$ = 0.01.

The evolution of the dispersed bubbles calculated using the Euler–Lagrange simulations is shown in Figure 3a and the 1% volume fraction iso-surface simulated with the Euler–Euler approach is shown in Figure 3b. The bubbles rise, driven by buoyancy. After their release from the sparger below the impeller, they get captured behind the impeller blades and detach from the upper edge of the impeller blades. After having passed the rear part of the impeller blades, the path of the rising bubbles is unimpeded in a vertical direction. However, due to drag, bubbles are swept along with the rotational motion of the liquid, resulting in upwards-directed spiraling bubble trajectories around the center of the vessel, which can be seen in both modeling approaches. Near the impeller, the volume fraction iso-surface of the Euler–Euler results is inclined in a clockwise direction because the anti-clockwise motion of bubbles is slower than the rotational velocity of the impeller, which is modeled with the rotating reference frame for the MRF region shown as blue parts in Figure 1. Above the rotating reference frame, the expected anti-clockwise inclination of the bubble plume is observed. The bubble dispersion and the residence time have a strong impact on the volumetric oxygen mass transfer coefficient, which will be discussed in Section 3.3.2. Another important aspect is the shape of the liquid surface addressed next.

### 3.2. Liquid Height, Surface Vortex Formation, and Risk of Cell Damage

**Figure 4:**
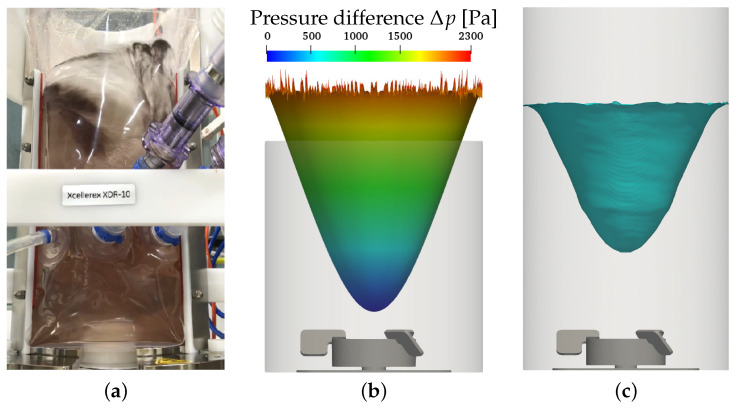
Surface vortex formation for 7.0 L, 350/360 rpm, 0.25 L min${}^{-1}$, 2 μm, experiment without sparging. (**a**) Experiment, 350 rpm; (**b**) EL results, 360 rpm; (**c**) EE results, $\alpha_{l}$ = 0.5, 360 rpm.

Since the Xcellerex${}^{\mathrm{TM}}$ XDR-10 is an unbaffled reactor with a centered impeller, changes in the liquid height due to surface vortex formation occur, especially at high impeller speeds. For the maximum impeller speed at the intermediate working volume (condition 8), surface vortex formation is seen in the experiments. Figure 4a shows a photo of the XDR-10 with an opened vessel frame. In the upper part, the slightly more transparent region in the top center of the liquid shows the surface vortex. The shadowed region to the left of the transparent region displays the left edge of the vortex. The white bar dividing the photo horizontally is part of the vessel frame. The white objects at the bottom center inside and below the inner bag are the cylindrical base of the impeller and the magnetic coupling driving the impeller. The Euler–Euler simulations reveal similar surface vortex formation shown as the 50% volume fraction iso-surface at the maximum impeller speed (see Figure 4c). In the Euler–Lagrange results, a high pressure gradient between the center and the edge of the top boundary is found, also indicating that strong surface vortex formation must be expected at the maximum impeller speed, see Figure 4b. The observation of the surface vortex correlates well with the measured changes in liquid height at the vessel wall, which are reported in Table 2. A strong surface vortex formation is found for the maximum impeller speed, whereas for all other conditions, the increase in liquid height remains at low values of up to 4 mm.

**Figure 5:**
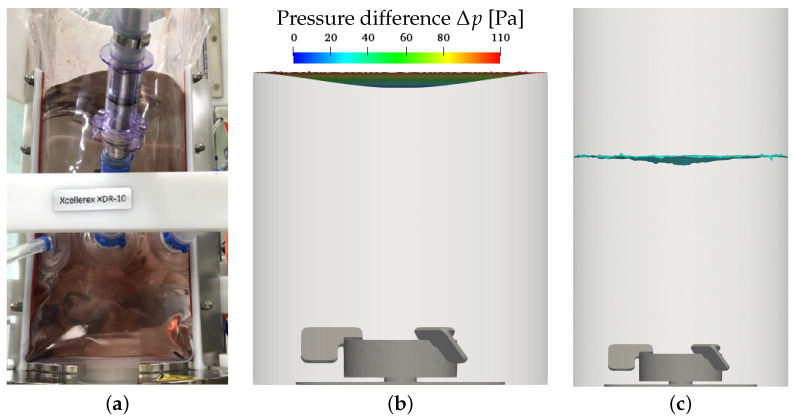
Surface vortex formation for 7.0 L, 100 rpm, 0.25 L min${}^{-1}$, 2 μm, experiment without sparging. (**a**) Experiment; (**b**) Euler–Lagrange results; (**c**) Euler–Euler results, $\alpha_{l}$ = 0.5.

The results for the reduced impeller speed of 100 rpm for the intermediate working volume of 7 L and for a sparging rate of 0.25 L min${}^{-1}$ and a pore size of 2 μm (base case) is shown in Figure 5. A slight liquid surface deformation is found in the experiments displayed in Figure 5a and in the Euler–Euler simulations, see Figure 5c. This also reflects in the results given in Table 2. Conditions 3–6 with different sparging rates and sparger types but the same impeller speed and working volume have the same vortex depth, see Table 2).

In the Euler–Lagrange simulations, the pressure gradient across the top boundary is low, also indicating slight liquid surface deformation (see Figure 5b). A similar observation is made for the lowest working volume of 4.5 L (condition 1, see Table 2). For the maximum working volume of 10 L (condition 2) and for the minimum impeller speed of 40 rpm (condition 7), the liquid height is not affected by stirring (see Table 2). For these two conditions, the liquid velocity close to the liquid surface is lowest and the velocity gradient from the center towards the vessel wall is too small to drive surface vortex formation.

**Figure 6:**
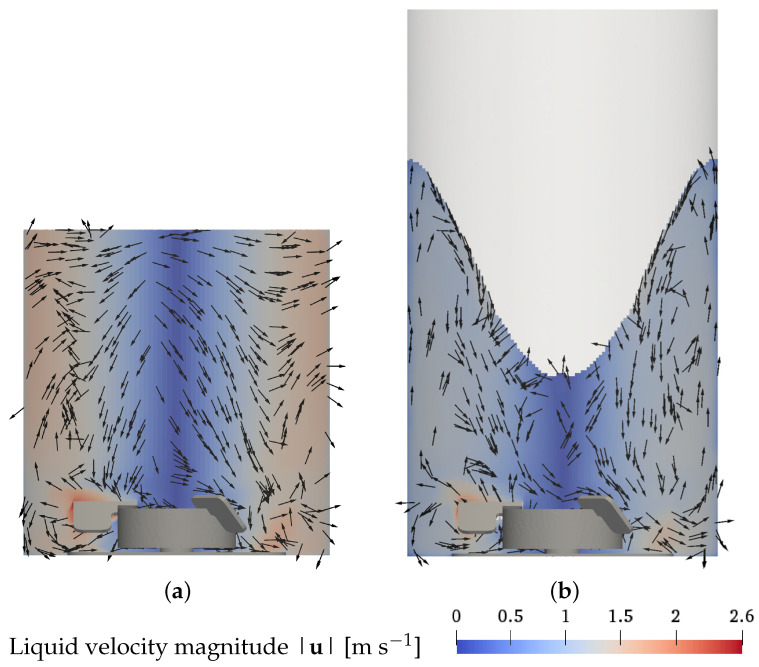
Liquid flow pattern for the maximum impeller speed condition (7 L, 360 rpm, 0.25 L min${}^{-1}$, 2 μm) in the *x*-*z* plane through the center of the vessel. Arrows indicate the liquid velocity in *x*-*z* direction. (**a**) Euler–Lagrange results; (**b**) Euler–Euler results.

The change in liquid height at the wall is less than a third of the total depth of the surface vortex in the Euler–Euler simulations (see Table 2). The same parametric dependence of the surface vortex depth on the operating conditions is evident in the Euler–Lagrange simulations. In the Euler–Lagrange approach, the liquid surface is represented by the flat top boundary and, accordingly, the possible surface vortex cannot be captured, whereas the Euler–Euler simulations directly reveal the surface vortex and the alteration of the shape of the upper recirculation zone (see Figure 6). Surface vortex formation can have a negative impact on cell growth [13,14,15] and, thus, running cell cultivation at extreme impeller speeds is not recommendable.

Different parameters exist for quantifying the hydrodynamic stress in the liquid phase. An easily accessible parameter is the volumetric power input, which linearly correlates to the average turbulence energy dissipation rate. Sieck et al. [16] report that negative effects on the productivity of Chinese hamster ovary (CHO) cells and transcriptomic stress responses are observed at an average turbulence energy dissipation rate of as low as 0.4 m${}^{2}$s${}^{-3}$. According to both simulation approaches, this level is exceeded for the maximum impeller speed (condition 8). Interpolating the volume average turbulence energy dissipation rate as a linear function of the impeller speed of 40, 100, and 360 rpm (conditions 7, 4, and, 8, respectively) suggests an upper limit of about 280 rpm to keep the average turbulence energy dissipation rate below the critical value reported by Sieck et al. [16]. In the Euler–Lagrange simulation, the average turbulence energy dissipation rate of 0.5 m${}^{2}$s${}^{-3}$ at 360 rpm (condition 8) is lower than the value of 0.7 m${}^{2}$s${}^{-3}$ observed in the Euler–Euler simulation. However, the Euler–Lagrange result is considered less reliable as the altered shape of the liquid volume due to the vortex formation is not captured and thus, the region of lower velocity and turbulence in the center of the vessel is larger, and consequently, the average turbulence energy dissipation rate is lower. The described discrepancy in the simulated average turbulence energy dissipation rate emphasizes that the Euler–Lagrange approach cannot adequately capture the flow for conditions with vortex formation.

**Figure 7:**
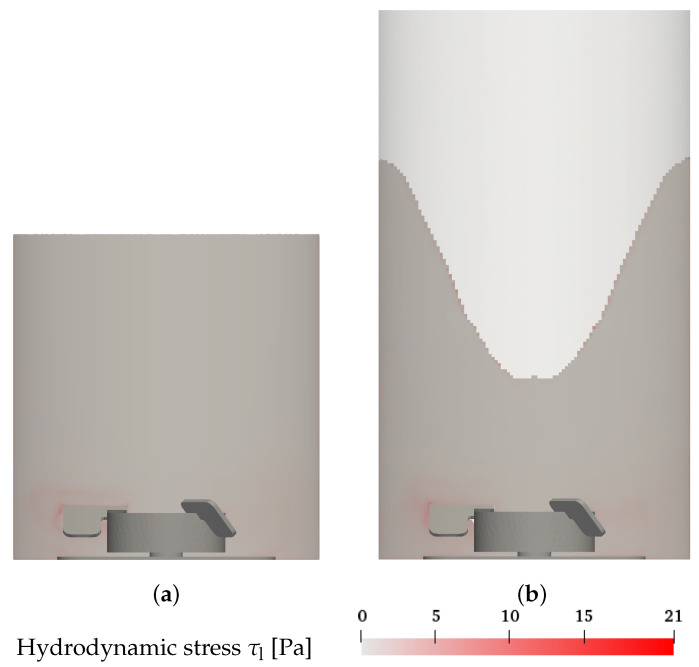
Hydrodynamic stress for the maximum impeller speed condition (7 L, 360 rpm, 0.25 L min${}^{-1}$, 2 μm) in the *x*-*z* plane through the center of the vessel. (**a**) Euler–Lagrange results; (**b**) Euler–Euler results.

While the average turbulence energy dissipation rate can be used as a criterion for hydrodynamic stress, the values of the turbulence energy dissipation rate show strong variation across different regions in the bioreactor and the maximum values can be multiple times higher than the average. Therefore, the local maximum values also must be considered to accurately account for the risk of cell damage. Soos et al. [17] calculated the local hydrodynamic stress caused by turbulent eddies as $\tau_{l}=\sqrt{\mu \rho \varepsilon }$ for which the highest values of hydrodynamic stress are found in the region of high velocity and high turbulence close to the impeller (see Figure 7). In the Euler–Lagrange simulations shown in Figure 7a and the Euler–Euler results displayed in Figure 7b, there are no significant differences in the results of hydrodynamic stress.

**Figure 8:**
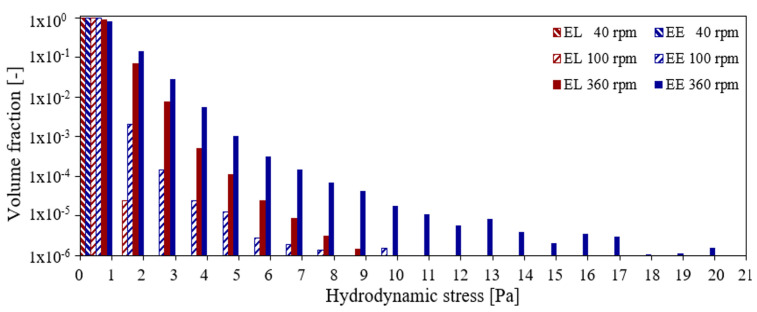
Liquid volume fraction distribution of the hydrodynamic stress regions for conditions 7, 4, and 8 for the EL and the EE simulations.

The distribution of the volume fraction of the different hydrodynamic stress levels is presented in Figure 8, where the volume fractions below 10${}^{-6}$ are excluded since their contribution is minor. The volume fraction distribution of the hydrodynamic stress supports that only a very small fraction of the liquid volume around the impeller blades (see Figure 7) and shows high hydrodynamic stress, while the bulk shows low hydrodynamic stress. Furthermore, in agreement with Christi [14], the maximum hydrodynamic stress strongly depends on the impeller speed (see Figure 8). Across all conditions, the Euler–Euler simulations calculate slightly higher hydrodynamic stress levels than the Euler–Lagrange simulations. This is likely associated with the turbulence induced by the bubbles which results in hydrodynamic stress due to sparging [31]. To further interpret the hydrodynamic stress results, the cell line-specific tolerance must be evaluated. Neunstoecklin et al. [18] reported a reduction in growth and productivity for maximum hydrodynamic stress values above 32.4 ± 4.4 Pa and 25.2 ± 2.4 Pa, respectively, for CHO and mouse hybridoma (Sp2/0) cells. For both simulation approaches, the hydrodynamic stress remains below these values for all conditions as shown in Figure 8.

**Figure 9:**
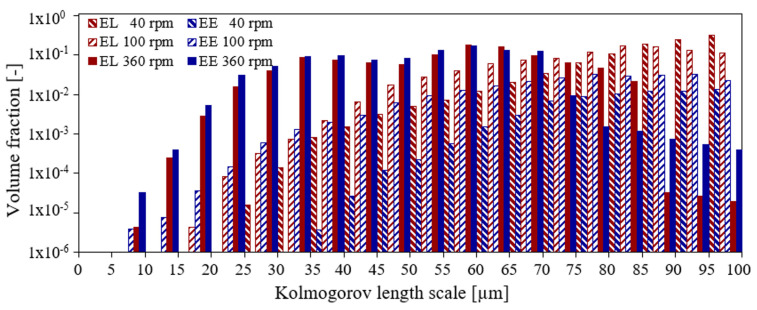
Liquid volume fraction distribution of the Kolmogorov length scale up to 100 μm for conditions 7, 4, and 8 for the EL and the EE simulations.

The Kolmogorov length scale $l_{K}$ may be evaluated from the numerical simulations, $l_{K}=\;{\left(\upsilon^{3}/\varepsilon \right)}^{0.25}$ or directly from the hydrodynamic stress as $l_{K}=\nu {\left(\rho /\tau_{l}\right)}^{1/2}$. Since the kinematic viscosity and the density are constant, the Kolmogorov length scale is inversely proportional to the square root of the hydrodynamic stress. The Kolmogorov length scale may be related directly to the size of cells, and Figure 9 allows for a direct evaluation of suitable operation conditions. The range of hydrodynamic stress from 1 Pa to 20 Pa corresponds to Kolmogorov length scales of up to 35.5 μm, and the scale is cut at 100 μm corresponding to a hydrodynamic stress of about 0.125 Pa. Figure 8 and Figure 9 reflect the inverse quadratic relation between the Kolmogorov length scale and the hydrodynamic stress.

The limit of 25.2 ± 2.4 Pa [18] of the maximum hydrodynamic stress for mammalian cells corresponds to a Kolmogorov length of about 7 μm. However, Kaiser et al. [4] report typical sizes of mammalian cells ranging between 15 μm and 20 μm. As the Kolmogorov length should not be smaller than the cell size, the Kolmogorov length of 7 μm might cause cell damage to typical mammalian cells. The operational impeller speed, considering the study of Neustoecklin et al. [18], ranges up to 360 rpm and that evaluated from the critical Kolmogorov length scale is 100 rpm as can be seen from Figure 9. This difference shows that experimental studies of the Kolmogorov length (or the turbulence dissipation rate) might be useful to clarify this discrepancy.

The different criteria for optimum cell cultivation can be summarized as follows: The maximum value for the tolerable maximum impeller speed based on the surface vortex formation is below 350 rpm and up to 280 rpm based on the average turbulence energy dissipation rate, and no limit is found with respect to the maximum hydrodynamic stress. Considering the Kolmogorov length scale would limit the maximum impeller speed to 100 rpm. Unfortunately, there is no experimental verification of the impact of the Kolmogorov length on cell cultivation available in literature yet. Thus, for a careful process design, multiple criteria have to be fulfilled.

### 3.3. Experimental and Numerical Mixing Time and Volumetric Oxygen Mass Transfer Coefficient

In this section, the experimental and numerical results for the mixing time and oxygen mass transfer coefficient are presented and discussed. All experimental data are reported terms of their mean value and the standard deviation evaluated from three experimental verifications.

#### 3.3.1. Mixing Time

**Figure 10:**
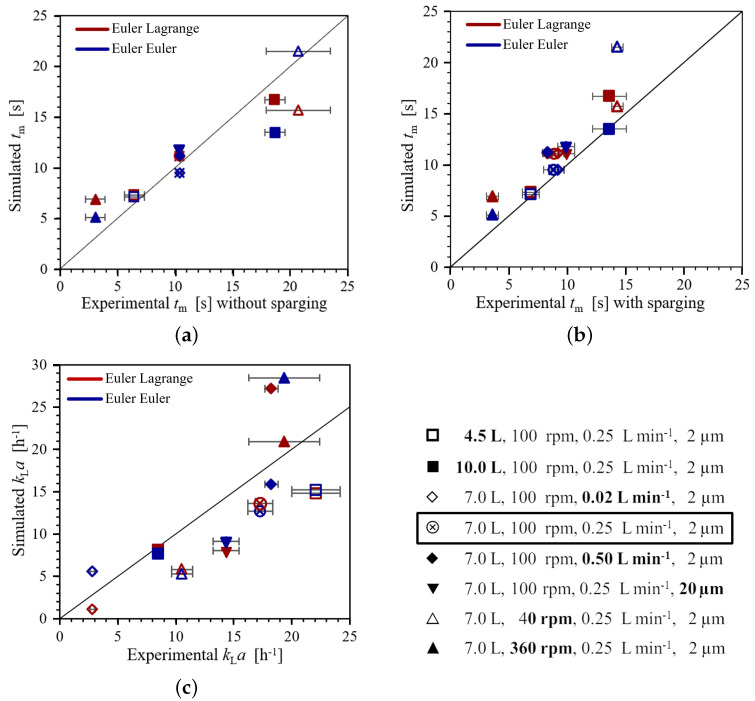
Experimental and simulation results of mixing times without and with sparging and for the volumetric oxygen mass transfer coefficient. (**a**) Experiments without sparging; (**b**) experiments with sparging; (**c**) volumentric oxygen mass transfer coefficient.

The experimental range of mixing times $t_{m}$ is 3.1 ± 0.8 s to 20.7 ± 2.8 s without sparging, and 3.6 ± 0.5 s to 14.3 ± 0.5 s with sparging (see Figure 10a,b). The shortest mixing times are found for the highest impeller speed (filled triangle) and the lowest working volume (open square), whereas the longest mixing times occur for the lowest impeller speed (open triangle) and the maximum working volume (filled square).

When comparing the experimental mixing times of conditions with sparging to those without sparging, the highest difference is found for the highest working volume and the lowest impeller speed with the longest mixing times, which are about 5 s and 6 s, respectively, longer without sparging. These results suggest that the liquid motion induced by sparging has a positive impact when mixing is slow but shows no effect when mixing is fast [32].

Evaluating the effect of different sparging rates (diamonds and circled cross in Figure 10a,b) and sparger types (circled cross and triangles down), the difference of 0.5–0.9 s between the conditions is of similar magnitude as the standard deviations of the individual conditions, and symbols for these conditions partially coincide in Figure 10a,b. Two-tailed Student’s *t*-tests for two samples [33] are used to evaluate the difference in the mean values of the different pairs of conditions with changes in the sparger type and sparging rate. The Student’s *t*-test results show no significant difference for a confidence interval of 95% for all pairs. Accordingly, the effects of the sparging rate and the sparger type on the mixing time are minor and too small to be verified with the standard deviation of the present number of measurements.

In Figure 10a,b, the simulated mixing times are compared to the experimental results without and with sparging. The same effects of the impeller speed and the working volume described for the experiments are found in the simulations (open and filled triangles and squares, respectively). The $R^{2}$ for the correlation of the experimental mixing times with sparging and the Euler–Euler simulations is 0.81, whereas the $R^{2}$ for the correlation in case of the Euler–Lagrange simulations is 0.92. Thus, in contrast to expectations, the simulations of the mixing times with sparging using the Euler–Euler approach, which captures the effect of bubbles on the liquid flow, is not superior to Euler–Lagrange simulations, which neglects the effect of the bubbles on the liquid. One reason for this might be the limitations of the measurement accuracy. Another reason might be that the difference between the two experimental data without and with sparging is minor for the majority of the conditions.

For the maximum impeller speed in Figure 10a,b (filled triangles), the Euler–Lagrange simulation over-predicts the mixing time, while the Euler–Euler simulation is closer to the experiment. This is most likely caused by the effect of surface vortex formation in the liquid flow, which is only captured in the Euler–Euler simulation and has significant impact on the liquid flow in the upper recirculation zone (see Section 3.2). Similar to experiments, changes in the sparging rate and sparger type also result in some minor variations in the mixing times simulated with the Euler–Euler approach, see Figure 10a,b (circled cross, diamonds, and triangles down).

Mishra et al. [10] also provide mixing time data for the XDR-10. They use the correlation proposed by Kaiser et al. [34] for a 3 L bioreactor to estimate the mixing times based on the volumetric power input. Mishra et al. [10] investigated impeller speeds between 50 rpm and 300 rpm, a working volume of 6.75 L, and sparging rates of 0.1 L min${}^{-1}$ and 0.2 L min${}^{-1}$. The closest conditions considered in the present study are a working volume of 7.0 L and a sparging rate of 0.25 L min${}^{-1}$. For impeller speeds of 50 rpm, 100 rpm, and 300 rpm, Mishra et al. [10] report mixing times of 13.9 s, 6.5 s, and 2 s, respectively, while in the present study, mixing times of 14.3 ± 0.5 s, 8.8 ± 0.9 s, and 3.6 ± 0.5 s are measured for impeller speeds of 40 rpm, 100 rpm, and 350 rpm. Thus, the agreement is good when comparing the results for 40 rpm and 50 rpm, but the difference increases for the higher impeller speeds. This indicates that the empirical correlation can provide a reasonable estimate, even though it is applied to a different bioreactor than the one for which it has originally been derived.

#### 3.3.2. Volumetric Oxygen Mass Transfer Coefficient

The experimentally observed range of the volumetric oxygen mass transfer coefficients $k_{L}a$ is 2.8 ± 0.3 h${}^{-1}$ to 22.1 ± 2.1 h${}^{-1}$, see Figure 10c.

The $k_{L}a$ increases with increasing impeller speed, see Figure 10 (triangles and circled cross) due to a higher $k_{L}$ and bubble residence time for increased liquid agitation as expected. However, the increase of 2.1 h${}^{-1}$ from the intermediate to the maximum impeller speed is too small to be significant because of the experimental standard deviations of 1.1 h${}^{-1}$ and 3.1 h${}^{-1}$ for the respective conditions as evaluated from Student’s *t*-test. Higher sparging rates, see Figure 10 (diamonds and circled cross), also result in a higher $k_{L}a$ because of an increased number of bubbles. Again, the increase of 0.9 h${}^{-1}$ from the intermediate value to the maximum value is too small to be significant. This may partially be due to the slightly higher standard deviation for the base condition and for the maximum impeller speed condition as well as possible experimental errors.

However, the reduced differences between the base condition 4 and the respective maximum levels of impeller speed and the sparging rate are smaller than those between the base case and the respective minimum levels of impeller speed and sparging rate. This indicates that for higher impeller speed and sparging rate, their positive impact on $k_{L}a$ diminishes. This effect is likely related to a higher probability of bubble coalescence for a higher gas hold-up and the reduced residence time of larger (coalesced) bubbles. While the simulations do not include coalescence, this physical process takes place inside the bioreactor and thus, it must be considered when evaluating the experimental results.

Higher working volumes, see Figure 10 (squares and circled cross), conform to lower volumetric power inputs, which result in a lower $k_{L}$ and consequently also a lower $k_{L}a$. A larger sparger pore size, i.e. 20 μm compared to 2 μm for the base case, leads to a significantly lower $k_{L}a$ due to a larger mean bubble diameter, see Figure 10 (triangles down). Accordingly, a smaller sparger pore size is preferable because it allows achieving the required $k_{L}a$ at a lower sparging rate.

For the simulation of the $k_{L}a$, the specific interface area and hence the bubble diameter is a critical parameter. If the bubble diameter considered in the simulations represents the actual Sauter mean diameter of the bubble size distribution well enough, the simulations should be able to accurately calculate the available oxygen mass transfer area. However, bubble sizes are subject to change not only due to different sparger types but also due to changes in the operating conditions.

In the present study, the bubble diameter is set according to the sparger type, and changes in bubble diameter due to other operating parameters are neglected. Accordingly, the simulations can only provide a rough estimate of $k_{L}a$ and the $R^{2}$ values of 0.62 and 0.53 for Euler–Lagrange and Euler–Euler simulations are low. For the majority of the conditions, the simulated $k_{L}a$ is lower than the experimental results indicating that the selected bubble diameter of 0.8 mm for the 2 μm sparger is too large for these conditions. Nevertheless, the same qualitative effects of changes in the operating conditions on the $k_{L}a$ as in the experiments are found: a higher sparging rate and impeller speed increase the $k_{L}a$, see Figure 10c (diamonds and triangles), while a higher working volume and larger sparger pore size reduce the $k_{L}a$, see Figure 10c (squares and triangles down).

The Euler–Lagrange and Euler–Euler simulations give $k_{L}a$ different values for the maximum impeller speed condition, see Figure 10c (filled triangle), since the simulated liquid flow differs due to the modeling of the surface vortex. The average turbulence energy dissipation rate is lower in the EL simulation compared to the EE results as discussed in Section 3.2, and accordingly, the liquid transfer coefficient, $k_{L}$, is lower than in the Euler–Euler simulation. Furthermore, the bubble residence time is longer in the Euler–Euler simulation than in the Euler–Lagrange simulation as the dispersion of the bubbles is stronger. These two effects result in a higher $k_{L}a$ in the Euler–Euler simulations. Furthermore, both the Euler–Lagrange and the Euler–Euler approaches predict a higher $k_{L}a$ than the experimental value for the considered bubble diameter of 0.8 mm (20.9 h${}^{-1}$ and 42.9 h${}^{-1}$ instead of 19.4 h${}^{-1}$, respectively). Since under this condition, the bubbles are strongly entrained into the impeller region, the mean bubble diameter is expected to increase due to coalescence. When the Euler–Euler simulation for this condition is repeated with a bubble diameter of 1 mm, a $k_{L}a$ of 28.5 h${}^{-1}$ is found. While this is still higher than the experimental value, it should also be kept in mind that $k_{L}$ is highest for this condition and accordingly, any over-prediction of the specific interface area *a* has a stronger effect on the $k_{L}a$ for this condition than for the other investigated operating conditions.

In a recent study, Mishra et al. [10] used population balance modeling to investigate the bubble size distribution and $k_{L}a$ for the 20 μm sparger. However, even with this more sophisticated modeling approach, deviations of up to 10% between measured and simulated $k_{L}a$ values are reported. However, even higher deviations between the experimental and the simulated values of the $k_{L}a$ are found in the literature [34,35,36]. Furthermore, Mishra et al. [10] reported experimental $k_{L}a$ values in the range of 2.5 h${}^{-1}$ to 5.5 h${}^{-1}$, which are significantly lower than the value of 14.9 h${}^{-1}$ observed for the 20 μm sparger in the present study. For this deviation, the exact reasons are unknown, but they are likely related to the differences in the experimental setup, i.e., Mishra et al. [10] did not apply head space airflow and start the stirring at the same time as the oxygen sparging instead of keeping it running during both the oxygen removal and the oxygen sparging steps.

In summary, in the present study, the qualitative effects of changes in the operating conditions are captured correctly, however, the accurate quantitative prediction of the $k_{L}a$ remains a challenge. This is partly due to the lack of data on the true bubble size distribution and the high standard deviation and potential errors in the experimental measurements of the $k_{L}a$. From the simulation point of view, the $k_{L}a$ is a complex process characteristic, which depends on multiple modeling aspects, including the bubble size and the velocity distribution as well as the liquid transfer coefficient $k_{L}$. If the eddy cell model [29] is used as in the present investigation, the $k_{L}$ is dependent on the turbulence energy dissipation rate of the liquid phase, and thus, the $k_{L}a$ is affected by both the gas and liquid phases. Therefore, experimental verification of the $k_{L}a$ is still needed. However, while recent studies work on improving model accuracy by including the bubble size distribution with population balance modeling [10,37,38], the single size assumption of the present study is suitable to account for the effects of changes in the operating conditions on the $k_{L}a$ and give relevant insight for the selection of the process conditions.

## 4. Conclusions

Both the Euler–Euler (EE) and Euler–Lagrange (EL) approaches can successfully predict the effect of different operating conditions on the hydrodynamic characteristics of the single-use bioreactor Xcellerex${}^{\mathrm{TM}}$ XDR-10. Surface vortex formation through either direct modeling in the EE or indirectly through the pressure profile at the top boundary in the EL model as well as the mixing time are simulated reasonably well. For the oxygen mass transfer coefficient, the overall effects of changes in operating conditions are captured with both modeling approaches even though the impact of the operating conditions on the bubble size is not considered. However, only the Euler–Euler approach can capture the influence of the surface vortex on the liquid flow at high impeller speeds.

For the selected conditions, only the maximum impeller speed indicates possible cell damage due to a high average turbulence energy dissipation rate and surface vortex formation. Accordingly, increasing the impeller speed to achieve a turbulence energy dissipation rate slightly below 0.4 m${}^{2}$s${}^{-3}$ (about 280 rpm based on an interpolation of the Euler–Euler simulation results) appears to be the upper limit of recommendable impeller speed for the Xcellerex${}^{\mathrm{TM}}$ XDR-10. High impeller speeds, but still below the limit of cell damage by hydrodynamic stress, are advantageous because they result in fast mixing and good oxygen mass transfer. However, based on the observed mixing times and volumetric oxygen mass transfer coefficients, an impeller speed of 100 rpm can already provide suitable cell culture conditions and at the same time, surface vortex formation is avoided.

It is concluded that the single-use XDR-10 bioreactor appears well suited to maintain hydrodynamic conditions suitable for animal cell culture despite the centered, bottom-mounted impeller and the unbaffled vessel, which is in contrast to classic bioreactor designs.

Since the cell density and oxygen uptake rates during animal cell cultivation are considerably lower than for microbial cells, the required oxygen mass transfer and the corresponding sparging rates are also low. Accordingly, the effect of the gas bubbles on the liquid flow is small and both the Euler–Lagrange and the Euler–Euler approaches give similar results for most conditions. However, this observation is also dependent on the considered operating conditions and if the relative impact of sparging on the liquid flow increases, i.e., for higher sparging rates or at very low impeller speeds, the ability of the one-way coupled Euler–Lagrange approach to characterize the flow correctly diminishes. Furthermore, the impact of surface vortex formation on the flow cannot be captured with the Euler–Lagrange approach and the use of the more advanced Euler–Euler model is required. Even though only the Euler–Euler approach accounts for the full interaction of both phases, the one-way coupled Euler–Lagrange simulations already provide good results at a significantly shorter computational time of less than 10% of the time required for the Euler–Euler simulations. Accordingly, the Euler–Lagrange approach can be useful for a first screening of potential operating conditions.

## Figures and Tables

**Table 1:** Operating conditions, ${}^{🟉}$ base case.

Condition	Working Volume	Impeller Speed	Sparging Rate	Sparger Pore Size
#	(L)	(rpm)	(L min${}^{-1}$)	(μm)
1	4.5	100	0.25	2
2	10.0	100	0.25	2
3	7.0	100	0.02	2
4 ${}^{☆}$	7.0	100	0.25	2
5	7.0	100	0.50	2
6	7.0	100	0.25	20
7	7.0	40	0.25	2
8	7.0	350/360	0.25	2

**Table 2:** Change in liquid height and vortex depth found in experiments (exp.), in Euler–Lagrange (EL), and in Euler–Euler (EE) simulations.

#	Condition	Change in Liquid Height		Vortex Depth	
		(mm)		(mm)	
		Exp.	EE	EE	EL
1	4.5 L, 100 rpm, 0.25 L min${}^{-1}$, 2 μm	4	2	10	15
2	10.0 L, 100 rpm, 0.25 L min${}^{-1}$, 2 μm	0	0	1	1
3	7.0 L, 100 rpm, 0.02 L min${}^{-1}$, 2 μm	3	1	8	12
4	7.0 L, 100 rpm, 0.25 L min${}^{-1}$, 2 μm	3	1	8	12
5	7.0 L, 100 rpm, 0.50 L min${}^{-1}$, 2 μm	3	1	8	12
6	7.0 L, 100 rpm, 0.25 L min${}^{-1}$, 20 μm	3	1	8	12
7	7.0 L, 40 rpm, 0.25 L min${}^{-1}$, 2 μm	0	0	0	1
8	7.0 L, 360 * rpm, 0.25 L min${}^{-1}$, 2 μm	28	44	145	229

* 350 rpm in experiments.

## Data Availability

Data available on request.
